# Draft genome of an extended-spectrum β-lactamase-producing multidrug-resistant *Escherichia coli* sequence type 131 strain isolated from the Msunduzi River in South Africa

**DOI:** 10.1128/mra.00136-25

**Published:** 2025-07-31

**Authors:** Nkosinathi D. Ndlazi, Maike Claussen, Stefan Schmidt

**Affiliations:** 1 Discipline of Microbiology, School of Life Sciences, University of KwaZulu-Natal 225515, Pietermaritzburg, South Africa; University of Maryland School of Medicine, Baltimore, Maryland, USA

**Keywords:** *Escherichia coli*, multidrug resistance, ESBL, river water, South Africa

## Abstract

The genome (length 4,949,272 bp, G+C content 50.70%) of the multidrug-resistant *Escherichia coli* strain 11.2.3 isolated from the Msunduzi River (South Africa) enabled assignment to sequence type 131, phylogroup B2, and serotype O16:H5. Antibiotic resistance genes (e.g., *bla*_CTX-M15_) and extraintestinal pathogenic *E. coli-*associated virulence factors (e.g., *fyuA*) were detected.

## ANNOUNCEMENT

*Escherichia coli* strains of sequence type (ST) 131 are well-known extraintestinal pathogenic *E. coli* (ExPEC), causing human blood and urinary tract infections (1–3). Strains of the pandemic clonal lineage ST131 frequently exhibit resistance against clinically relevant antibiotics (e.g., β-lactams, fluoroquinolones) and produce extended-spectrum-β-lactamases (ESBL) (1, 2, 4, 5). They are usually associated with humans or animals, but were also detected in waste or river water (2, 3, 5, 6).

**Fig 1:**
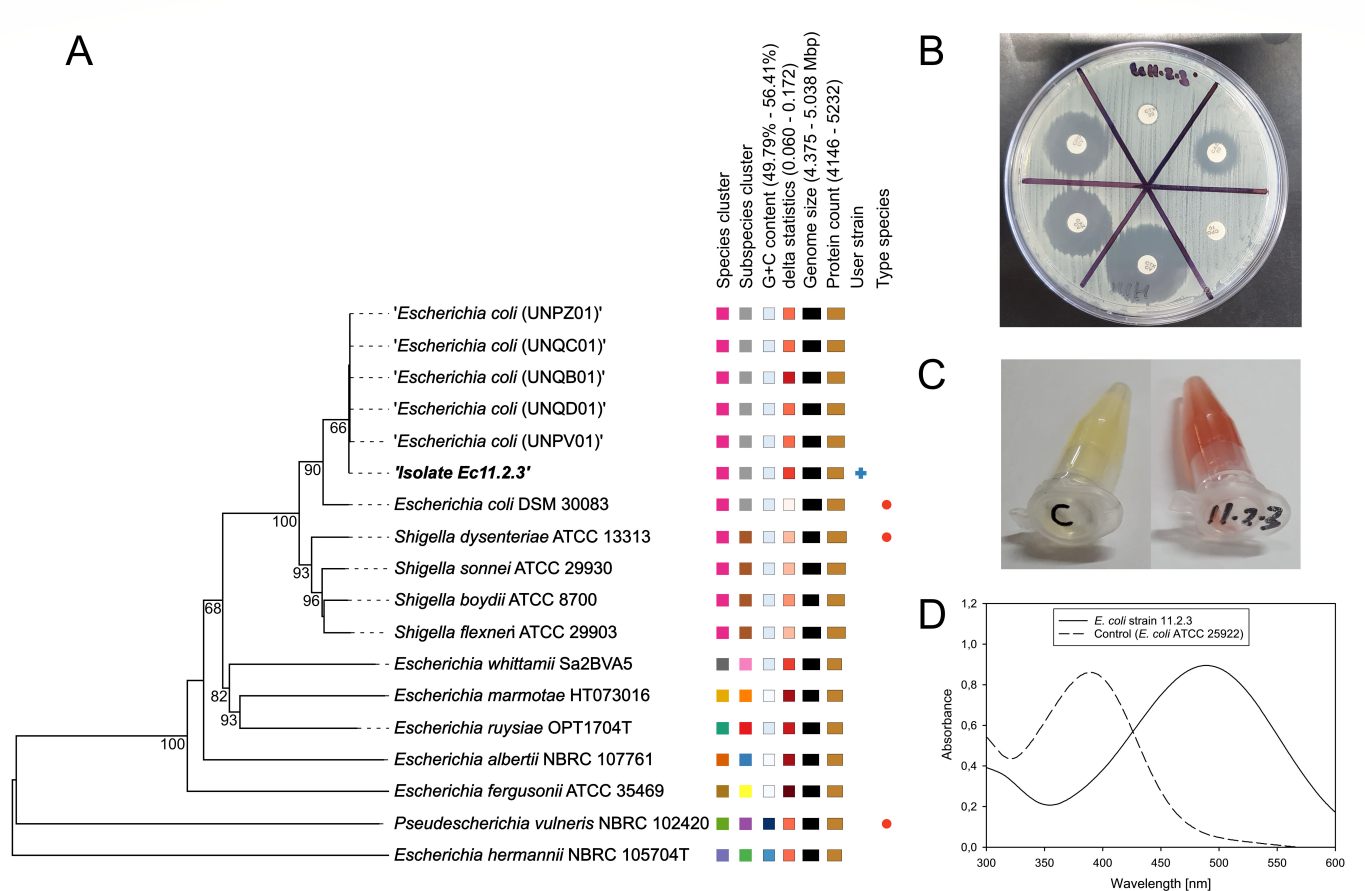
Phylogenomic comparison of the multidrug-resistant, ESBL-producing *E. coli* strain 11.2.3 and representative antibiotic resistance-related strain features. (**A**) Midpoint-rooted phylogenetic tree inferred with FastME (2.1.6.1) from genome blast distance phylogeny (GBDP) distances calculated from type and reference strain genome sequences using the Type Strain Genome Server (TYGS) and default parameters. The five most closely related (ANIb) genomes of *E. coli* ST131-O16:H5 strains were additionally included. Branch lengths are scaled based on the GBDP distance formula d5; numbers shown are GBDP pseudo-bootstrap support values ≥ 60% (100 replications, average branch support = 60.7%). (**B**) *E. coli* strain 11.2.3 exhibiting an ESBL phenotype with enlarged zones of inhibition for three representative third-generation cephalosporins (ceftazidime, cefotaxime, and cefpodoxime) in the presence of the ESBL inhibitor clavulanate compared to matching cephalosporin-only disks when using the MAST D67C ESBL test. (**C**) β-Lactam ring hydrolysis of the chromogenic cephalosporin nitrocefin (yellow) to yield the red hydrolysis product by cells of strain 11.2.3 but not cells of a negative control (C = *E. coli* ATCC 25922). (**D**) Spectroscopic demonstration of β-lactam ring hydrolysis of the chromogenic cephalosporin nitrocefin by cells of *E. coli* strain 11.2.3. Negative control (broken line, *E. coli* ATCC 29522, λ_max_~390 nm, yellow) and ESBL phenotype *E. coli* strain 11.2.3 (solid line, λ_max_~486 nm, red). The resulting bathochromic shift was captured by recording spectra (cell OD_600_ = 1 in 50 mM phosphate buffer, pH 7, 30 min incubation at 37°C, 50 µM nitrocefin) using cell-free supernatant.

*E. coli* strain 11.2.3 was isolated in 2022 when analyzing water samples collected from the Msunduzi River in Pietermaritzburg (KwaZulu-Natal, South Africa) for the presence of *E. coli*, employing a well-established MPN standard method (7) followed by confirmation using an *E. coli*-specific diagnostic amplicon PCR targeting the *gadA* gene (8). Antibiotic susceptibility testing using the EUCAST disk diffusion procedure (V14, https://www.eucast.org/fileadmin/src/media/PDFs/EUCAST_files/Breakpoint_tables/v_14.0_Breakpoint_Tables.pdf) and the MAST D67C ESBL kit revealed that the strain was multidrug-resistant, exhibiting an ESBL phenotype (Table 1; Fig. 1). Additionally, cells of strain 11.2.3 hydrolyzed the β-lactam ring of the chromogenic cephalosporin nitrocefin to the expected red reaction product, verified by spectrophotometric analysis against a negative control (Fig. 1) (9).

**TABLE 1:** Genome characteristics of an ESBL-producing multidrug-resistant *E. coli* strain from the Msunduzi River in South Africa

Characteristic	Detail
Strain and basic features	*E. coli* strain 11.2.3, rod-shaped Gram-negative motile cells, oxidase-negative, gas produced from lactose at 44.5°C, indole produced from tryptophan, positive methyl red test, and β-glucuronidase positive
Isolation year, origin, location, province, and country	2022, Msunduzi River water sample, Pietermaritzburg, KwaZulu-Natal, and South Africa
GenBank accession number	JBJXCV000000000
Number of reads (input) and average short-read coverage	3 727 182 and 97
Number of contigs, largest contig, N50, and L50	115, 575 749 bp, 219 220 bp, and 9
Genome length and G+C content	4 949 272 bp and 50.70%
Completeness (BUSCO V5, enterobacterales_odb10)	100%
Protein coding sequences, tRNA, and rRNA	4821, 76, and 4
Identity (rMLST)	*E. coli* (100%)
Strain typing (sequence type^Enterobase/Pasteur^, phylogroup, serotype, and *fimH* type)	ST131/506, B2, O16:H5, and *fimH*41
Digital DNA:DNA hybridization (dDDH, d4), best type strain genome match	*E. coli* DSM 30083^T^ (GCF_000690815.1) (86%)
dDDH (d4), best ST131-O16:H5 strain genome match	*E. coli* ST131-O16:H5 UNQD01 (GCF_900536525.1) (99.70%)
ANIb best type strain genome match	*E. coli* DSM 30083^T^ (GCF_000690815.1) (98.11%)
ANIb best ST131-O16:H5 strain genome match	*E. coli* ST131-O16:H5 UNQD01 (GCF_900536525.1) (99.89%)
Antibiotic-resistance genes	*aac(3)-IId*, *aph(3'')-Ib*, *aph(6)-Id*, *bla*_CTX-M15_, *bla*_TEM-1B_, *dfrA17*, *mph*(A), *qacE*, *sul1*, *sul2*, and *tet*(A)
Known chromosomal mutations enabling antibiotic resistance	*gyrA* (p.S83L), *gyrA* (p.D87N), *parC* (p.S80I), and *parC* (p.E84V)
Phenotypic antibiotic resistance profile	Ampicillin, aztreonam, cefalexin, cefotaxime, ceftaroline, ceftazidime, ciprofloxacin, gentamicin, levofloxacin, moxifloxacin, norfloxacin, tobramycin, trimethoprim, and trimethoprim-sulfamethoxazole
Prophages	3
Plasmid replicon types detected	FIA, FIB, and FII
Human pathogen probability	92.7%
Representative virulence factor encoding genes associated with ExPEC pathotypes	*afaA/C/D*, *aslA*, *chuA*, *fimH*, *fyuA*, *iha*, *iucC*, *iutA*, *kpsE*, *kpsMII_K5*, *nlpI*, *ompT*, *sat*, *senB*, *sitA*, *traT*, and *yfcV*

DNA was extracted using the Quick-DNA miniprep kit (Zymo, USA) from washed cells harvested by centrifugation from an overnight culture (37°C, 100 rpm, Nutrient broth, Neogen). Before shotgun sequencing, DNA was quantified using a NanoDrop 2000 spectrophotometer, followed by preparing a library using the FS DNA Library Prep Kit with enzymatic fragmentation (NEB, USA) and sequencing (Illumina NextSeq) to establish 2 × 150 bp paired-end reads (Inqaba Biotech, South Africa). Raw reads were quality checked (FastQC V0.12.1), trimmed (TrimGalore V0.6.5, Cutadapt V4.2), assembled (Unicycler V0.4.8, 500 bp minimum contig size), polished (Pilon V1.23), and quality controlled (Quast V5.2.0) using the BV-BRC analysis pipeline (10–16). Annotation was done using PGAP (V6.9) (17). Antibiotic resistance and virulence genes, plasmid replicons, and prophages were detected using ResFinder 4.6, VirulenceFinder 2.0, PlasmidFinder 2.1, and Phigaro 2.0 (18–21). Default parameters were used for all software tools unless otherwise specified.

The genome of strain 11.2.3 (4,949,272 bp) was 100% complete according to the benchmarking tool BUSCO V.5 (22). The essential genomic features are summarized in Table 1. Multilocus sequence (PubMLST-tools) (23) and additional strain typing tools (ECTyper 2.0, ClermonTyper 22.03, FimTyper 1.0, and PathogenFinder 2.1) (24–27) confirmed strain 11.2.3 as *E. coli* ST131-B2-O16:H5-*FimH*41. Sequence similarity (ANIb) (28) and phylogenetic analyses using the TYGS-LPSN database with genome blast distance phylogeny and automatically selected reference genomes (29) demonstrated that strain 11.2.3 clusters with the five most similar (ANIb) genomes of *E. coli* ST131-O16:H5 isolates from France (Fig. 1). Furthermore, the strain exhibited an ExPEC-like virulence gene profile (Table 1) (19). Antibiotic resistance genes detected (e.g., *aph(6)-Id, bla*_CTX-M-15_, and *sul1*) generally matched the phenotypic antibiotic resistance profile of strain 11.2.3, with resistance against β-lactams (e.g., aztreonam and cefotaxime), fluoroquinolones (e.g., ciprofloxacin), aminoglycosides (e.g., tobramycin), and sulfonamide (e.g., trimethoprim-sulfamethoxazole) (Table 1).

Our results are concerning as third-generation cephalosporin-resistant *E. coli* are regarded as critical priority pathogens by the World Health Organization (30). Such ESBL multidrug-resistant pathogenic bacteria in the Msunduzi River are a potential health risk when the water is used for recreational, domestic, or agricultural purposes.

## Data Availability

This whole-genome shotgun project has been deposited at DDBJ/ENA/GenBank under the accession JBJXCV000000000. The version described in this paper is version JBJXCV00000000.1. The genome assembly was deposited under accession number GCF_046638585.1 (bioproject number PRJNA843922). The raw reads were deposited under accession number SRR31667184.

## References

[1] Banerjee R, Johnson JR. 2014. A new clone sweeps clean: the enigmatic emergence of Escherichia coli sequence type 131. Antimicrob Agents Chemother 58:4997–5004. doi:10.1128/AAC.02824-14 24867985 PMC4135879

[2] Denamur E, Clermont O, Bonacorsi S, Gordon D. 2021. The population genetics of pathogenic Escherichia coli. Nat Rev Microbiol 19:37–54. doi:10.1038/s41579-020-0416-x 32826992

[3] Kawamura K, Nagano N, Suzuki M, Wachino J-I, Kimura K, Arakawa Y. 2017. ESBL-producing Escherichia coli and its rapid rise among healthy people. Food Saf (Tokyo) 5:122–150. doi:10.14252/foodsafetyfscj.2017011 32231938 PMC6989193

[4] Ben Zakour NL, Alsheikh-Hussain AS, Ashcroft MM, Khanh Nhu NT, Roberts LW, Stanton-Cook M, Schembri MA, Beatson SA. 2016. Sequential acquisition of virulence and fluoroquinolone resistance has shaped the evolution of Escherichia coli ST131. mBio 7:e00347-16. doi:10.1128/mBio.00347-16 27118589 PMC4850260

[5] Gomi R, Matsuda T, Matsumura Y, Yamamoto M, Tanaka M, Ichiyama S, Yoneda M. 2017. Occurrence of clinically important lineages, including the sequence type 131 C1-M27 subclone, among extended-spectrum-β-lactamase-producing Escherichia coli in wastewater. Antimicrob Agents Chemother 61:e00564-17. doi:10.1128/AAC.00564-17 28630184 PMC5571296

[6] Sato T, Uemura K, Yasuda M, Maeda A, Minamoto T, Harada K, Sugiyama M, Ikushima S, Yokota S-I, Horiuchi M, Takahashi S, Asai T. 2024. Traces of pandemic fluoroquinolone-resistant Escherichia coli clone ST131 transmitted from human society to aquatic environments and wildlife in Japan. One Health 18:100715. doi:10.1016/j.onehlt.2024.100715 39010959 PMC11247291

[7] Health Canada. 2002. Enumeration of coliforms, faecal coliforms and of E. coli in foods using the MPN method. MFHPB-19. Ottawa, Canada.

[8] Gemmell ME, Schmidt S. 2013. Is the microbiological quality of the Msunduzi River (KwaZulu-Natal, South Africa) suitable for domestic, recreational, and agricultural purposes? Environ Sci Pollut Res 20:6551–6562. doi:10.1007/s11356-013-1710-1 23608984

[9] Ghavami A, Labbé G, Brem J, Goodfellow VJ, Marrone L, Tanner CA, King DT, Lam M, Strynadka NCJ, Pillai DR, Siemann S, Spencer J, Schofield CJ, Dmitrienko GI. 2015. Assay for drug discovery: synthesis and testing of nitrocefin analogues for use as β-lactamase substrates. Anal Biochem 486:75–77. doi:10.1016/j.ab.2015.06.032 26142222

[10] Andrews S. 2010. FastQC: a quality control tool for high throughput sequence data. Available from: http://www.bioinformatics.babraham.ac.uk/projects/fastqc/FASTQC

[11] Krueger F. 2019. Trim Galore: a wrapper tool around Cutadapt and FastQC to consistently apply quality and adapter trimming to FastQ files, with some extra functionality for MspI-digested RRBS-type (Reduced Representation Bisufite-Seq) libraries. Available from: http://www.bioinformatics.babraham.ac.uk/projects/trim_galore

[12] Martin M. 2011. Cutadapt removes adapter sequences from high-throughput sequencing reads. EMBnet J 17:10. doi:10.14806/ej.17.1.200

[13] Wick RR, Judd LM, Gorrie CL, Holt KE. 2017. Unicycler: resolving bacterial genome assemblies from short and long sequencing reads. PLoS Comput Biol 13:e1005595. doi:10.1371/journal.pcbi.1005595 28594827 PMC5481147

[14] Walker BJ, Abeel T, Shea T, Priest M, Abouelliel A, Sakthikumar S, Cuomo CA, Zeng Q, Wortman J, Young SK, Earl AM. 2014. Pilon: an integrated tool for comprehensive microbial variant detection and genome assembly improvement. PLoS One 9:e112963. doi:10.1371/journal.pone.0112963 25409509 PMC4237348

[15] Gurevich A, Saveliev V, Vyahhi N, Tesler G. 2013. QUAST: quality assessment tool for genome assemblies. Bioinformatics 29:1072–1075. doi:10.1093/bioinformatics/btt086 23422339 PMC3624806

[16] Olson RD, Assaf R, Brettin T, Conrad N, Cucinell C, Davis JJ, Dempsey DM, Dickerman A, Dietrich EM, Kenyon RW, et al.. 2023. Introducing the bacterial and viral bioinformatic resource center (BV-BRC): a resource combining PATRIC, IRD and ViPR. Nucleic Acids Res 51:D678–D689. doi:10.1093/nar/gkac1003 36350631 PMC9825582

[17] Tatusova T, DiCuccio M, Badretdin A, Chetvernin V, Nawrocki EP, Zaslavsky L, Lomsadze A, Pruitt KD, Borodovsky M, Ostell J. 2016. NCBI prokaryotic genome annotation pipeline. Nucleic Acids Res 44:6614–6624. doi:10.1093/nar/gkw569 27342282 PMC5001611

[18] Bortolaia V, Kaas RS, Ruppe E, Roberts MC, Schwarz S, Cattoir V, Philippon A, Allesoe RL, Rebelo AR, Florensa AF, et al.. 2020. ResFinder 4.0 for predictions of phenotypes from genotypes. J Antimicrob Chemother 75:3491–3500. doi:10.1093/jac/dkaa345 32780112 PMC7662176

[19] Malberg Tetzschner AM, Johnson JR, Johnston BD, Lund O, Scheutz F. 2020. In silico genotyping of Escherichia coli isolates for extraintestinal virulence genes by use of whole-genome sequencing data. J Clin Microbiol 58:e01269-20. doi:10.1128/JCM.01269-20 32669379 PMC7512150

[20] Carattoli A, Zankari E, García-Fernández A, Voldby Larsen M, Lund O, Villa L, Møller Aarestrup F, Hasman H. 2014. In silico detection and typing of plasmids using PlasmidFinder and plasmid multilocus sequence typing. Antimicrob Agents Chemother 58:3895–3903. doi:10.1128/AAC.02412-14 24777092 PMC4068535

[21] Starikova EV, Tikhonova PO, Prianichnikov NA, Rands CM, Zdobnov EM, Ilina EN, Govorun VM. 2020. Phigaro: high-throughput prophage sequence annotation. Bioinformatics 36:3882–3884. doi:10.1093/bioinformatics/btaa250 32311023

[22] Manni M, Berkeley MR, Seppey M, Simão FA, Zdobnov EM. 2021. BUSCO update: novel and streamlined workflows along with broader and deeper phylogenetic coverage for scoring of eukaryotic, prokaryotic, and viral genomes. Mol Biol Evol 38:4647–4654. doi:10.1093/molbev/msab199 34320186 PMC8476166

[23] Jolley KA, Bray JE, Maiden MCJ. 2018. Open-access bacterial population genomics: BIGSdb software, the PubMLST.org website and their applications. Wellcome Open Res 3:124. doi:10.12688/wellcomeopenres.14826.1 30345391 PMC6192448

[24] Bessonov K, Laing C, Robertson J, Yong I, Ziebell K, Gannon VPJ, Nichani A, Arya G, Nash JHE, Christianson S 2. 2021. ECTyper: in silico Escherichia coli serotype and species prediction from raw and assembled whole-genome sequence data. Microb Genom 7:000728. doi:10.1099/mgen.0.000728 34860150 PMC8767331

[25] Beghain J, Bridier-Nahmias A, Le Nagard H, Denamur E, Clermont O. 2018. ClermonTyping: an easy-to-use and accurate in silico method for Escherichia genus strain phylotyping. Microb Genom 4:e000192. doi:10.1099/mgen.0.000192 29916797 PMC6113867

[26] Roer L, Tchesnokova V, Allesøe R, Muradova M, Chattopadhyay S, Ahrenfeldt J, Thomsen MCF, Lund O, Hansen F, Hammerum AM, Sokurenko E, Hasman H. 2017. Development of a web tool for Escherichia coli subtyping based on fimH alleles. J Clin Microbiol 55:2538–2543. doi:10.1128/JCM.00737-17 28592545 PMC5527432

[27] Cosentino S, Voldby Larsen M, Møller Aarestrup F, Lund O. 2013. PathogenFinder - distinguishing friend from foe using bacterial whole genome sequence data. PLoS One 8:e77302. doi:10.1371/journal.pone.0077302 24204795 PMC3810466

[28] Richter M, Rosselló-Móra R, Oliver Glöckner F, Peplies J. 2016. JSpeciesWS: a web server for prokaryotic species circumscription based on pairwise genome comparison. Bioinformatics 32:929–931. doi:10.1093/bioinformatics/btv681 26576653 PMC5939971

[29] Meier-Kolthoff JP, Carbasse JS, Peinado-Olarte RL, Göker M. 2022. TYGS and LPSN: a database tandem for fast and reliable genome-based classification and nomenclature of prokaryotes. Nucleic Acids Res 50:D801–D807. doi:10.1093/nar/gkab902 34634793 PMC8728197

[30] World Health Organization (WHO). 2024. WHO bacterial priority pathogens list, 2024: bacterial pathogens of public health importance to guide research, development and strategies to prevent and control antimicrobial resistance. ISBN: 978-92-4-009346-1. World Health Organization, Geneva. http://www.who.int/publications/i/item/9789240093461.

